# Unaccompanied homeless youth have extremely poor diet quality and nutritional status

**DOI:** 10.1080/02673843.2018.1538885

**Published:** 2018-10-27

**Authors:** Irene Hatsu, Carolyn Gunther, Erinn Hade, Stephanie Vandergriff, Natasha Slesnick, Rachel Williams, Richard S. Bruno, Julie Kennel

**Affiliations:** a Human Nutrition Program, Department of Human Sciences, The Ohio State University, Columbus, OH, USA; b Center for Biostatistics, College of Medicine, The Ohio State University, Columbus, OH, USA; c Human Development and Family Science Program, Department of Human Sciences, The Ohio State University, Columbus, OH, USA

**Keywords:** Unaccompanied youth, homeless, nutritional status, diet quality

## Abstract

A lack of in-depth assessment of the nutritional status of homeless youth precludes interventions that achieve nutritional adequacy. We enrolled 118 unaccompanied homeless youth to obtain sociodemographic and health data along with dietary, anthropometric, biochemical, and clinical assessments. As a reference, homeless youth data were compared to a convenience sample of 145 college students. Obesity was prevalent among homeless youth than among college students (29% vs. 8% respectively (CI: 11.2, 29.9). Among homeless youth, 74% of females versus 41% of males were overweight/obese (CI: 14.9, 51.2). Homeless youth also had poor diet quality (44.37 (SD: 12.64)). Over 70% of homeless youth had inadequate intakes of vitamins A, C, D_3_ and E, as well as calcium and magnesium. Our findings show increased weight, adiposity, and suboptimal intakes of essential nutrients among unaccompanied homeless youth. Further studies are needed to inform evidence-based nutrition interventions that will aid in improving their nutritional health.

## Introduction

The transition from late adolescence to early adulthood is a period of nutritional vulnerability due to continued nutrient requirements for growth, poor eating patterns, risk taking behaviors, increased autonomy, and influence of environmental factors such as food insecurity (Delisle, 2005). Consequently, young adults are at risk for developing cardiometabolic abnormalities and chronic diseases such as obesity, diabetes, hypertension, and cardiovascular diseases associated with poor nutrition.

A group of young adults disproportionally affected by issues of inadequate nutrition are homeless youth (Gaetz, O’Grady, Buccieri, Karabanow, & Marsolais, 2013). Up to 3.5 million homeless individuals live in the U.S. (Burt, 2000; NLCHP, 2004) and about 1.3 million U.S. children and young adults experience homelessness in a given year (NCHE, 2015). Within the vulnerable homeless young adult population are a subset of individuals without a family or parental support, known as ‘unaccompanied’ homeless youth: they are a growing segment of the U.S. population and account for up to 7% of the total homeless population (Henry, Watt, Rosenthal, & Shivji, 2016). Unaccompanied youth are a difficult to reach population and hence the least understood in terms of their diet and nutritional health needs. This is particularly concerning given that they experience higher nutrition-related physical and mental health challenges compared to homeless youth with families and housed youth (Edidin, Ganim, Hunter, & Karnik, 2012).

The limited studies in this area of inquiry are among non-U.S. samples and demonstrate that homeless young adults have limited access to food and are failing to meet the basic requirements for essential nutrients (Tarasuk, Dachner, & Li, 2005; Tarasuk, Dachner, Poland, & Gaetz, 2009). It is evident that the adversities faced by unaccompanied homeless youth increase their risk for nutritional vulnerabilities; however, such vulnerabilities are yet to be systematically examined within the context of the United States. Furthermore, current interventions and services that address the diverse health needs of homeless youth in the US focus on housing, mental health services, alcohol and drug treatment and HIV/AIDS risk reduction, with limited emphasis on nutrition (Altena, Brilleslijper-Kater, & Wolf, 2010; Medlow, Klineberg, & Steinbeck, 2014; Slesnick, Dashora, Letcher, Erdem, & Serovich, 2009; Xiang, 2012). Improving the health of homeless youth requires a holistic approach that should include a focus on nutritional health (Kulik, Gaetz, Crowe, & Ford-Jones, 2011). Emerging evidence shows s relationship between poor nutritional status and several of the health challenges faced by homeless youth, especially mental health (Parletta, Milte, & Meyer, 2013; Rao, Asha, Ramesh, & Rao, 2008).

According to a systematic review on the health diagnosis of homeless youth, accurate data is needed on their physical health diagnosis, including that of their nutritional health (Medlow, et al., 2014). This information is critical to support the delivery of health interventions that span the breath of physical and mental health challenges presented in this population (Medlow, et al., 2014). In view of this, there is the need to investigate the dietary patterns and nutritional outcomes of unaccompanied homeless youth in the U.S. Without this knowledge, we will be precluded to develop effective and targeted health-promoting policy or programmatic interventions designed to address the nutritional vulnerability in this already at-risk group. The objective of this study was to provide an in-depth assessment of the dietary pattern and nutritional status of unaccompanied homeless young adults in a Midwestern state in the US. We hypothesized that these unaccompanied youth will have poor dietary and nutritional status based on established criteria indicative of optimal nutritional health. To address this hypothesis we conducted a comprehensive assessment of nutritional status, and also compared outcomes to findings obtained from a convenient sample of apparently healthy domiciled college students. Thus, the outcomes are expected to inform future research directions among vulnerable populations of homeless youth.

## Methods

### Study sample and design

The target population was unaccompanied homeless young adults receiving services from a dropin center in Columbus OH. Inclusion criteria were: 1) met homelessness criterion as defined according to the McKinney-Vento Act (2002): ‘those who lack a fixed, regular, and adequate nighttime residence’ (U.S.C, 2002); 2) were between the ages of 18–24 years; and 3) were willing to sign an informed consent. The only exclusion criterion was self-reported pregnancy, due to the changes that occur with body composition and dietary intake during this life stage. Ethical approval for this study was provided by the Institutional Review Board of the Ohio State University.

Potential homeless youth participants were identified through flyers and word of mouth. Youth were informed of study procedures, benefits, and risks and asked to sign a written informed consent. Following consent, each youth completed a screening questionnaire; those meeting eligibility criteria were included in the study. Although this was a cross-sectional study, data were collected at two different visits to reduce participation burden. The initial visit consisted of surveys assessing sociodemographic, as well as health status and homeless experience characteristics. In addition, anthropometric and biochemical assessments were completed along with a 24-hr dietary recall. During the second visit, participants completed a second 24-h dietary recall. All study activities and assessments were completed at the drop-in center. Each youth was provided a $15 grocery store and $10 restaurant gift card after completing all study requirements.

### Measures

#### Sociodemographic and health characteristics

A self-administered paper survey was used to gather data on demographic (age, gender, race/ ethnicity, education, employment status) as well as health status and homelessness characteristics (age at onset of homelessness, length of homelessness).

#### Anthropometric measurements

Measures of height and weight were assessed and used to determine body mass index (BMI). Height was measured to the nearest 0.1 centimeter using a stadiometer that had a vertical board and an adjustable headpiece and weight was measured to the nearest 0.1 kg using a calibrated digital scale. Waist circumference was measured to the nearest 0.1 cm and used to calculate waistto-height (WHtR) ratio. Waist circumference was assessed by placing an inelastic plastic tape measure snuggly around the midsection of a standing participant without compressing the skin, at the level of the iliac crest and read on the right side of the body (Wang, Thornton, Kolesnik, & Pierson, 2000). WHtR were calculated and compared to the global boundary of 0.5. Values above this cut-point indicate an increased cardiometabolic risk for CVD and diabetes (Browning, Hsieh, & Ashwell, 2010). Anthropometric measurements were carried out with participants wearing light clothing and no shoes.

#### Biochemical and clinical assessment

Non-fasting venous blood was obtained to assess serum concentrations of triglycerides, total cholesterol, low-density lipoprotein (LDL) cholesterol, and high-density lipoprotein (HDL) cholesterol (Nordestgaard et al., 2016). Non-fasting samples were utilized because the youth were noncompliant with a fasting protocol for lipid testing. Blood was drawn by a trained nurse according to a standardized protocol. Following a 30 minutes incubation period, to allow for clotting, the samples were centrifuged, separated and aliquots were stored at −80°C in cryovials. Defrosted serum samples were analyzed in singular using the Dimension Xpand Clinical Chemistry System (Siemens Medical Diagnostics, Decatur, Ga.). The analytical sensitivity for HDL, LDL, Cholesterol and Triglycerides were 3.0 mg/dL, 5 mg/dL, 50 mg/dL and 15 mg/dL respectively. Concentrations of triglycerides were considered elevated at ≥175mg/dl, total cholesterol at ≥190mg/dl, LDL cholesterol at ≥115mg/dl, and HDL cholesterol at ≤ 40mg/dl, according to guidelines for non-fasting samples (Nordestgaard et al., 2016). To measure blood pressure, participants assumed a seated position with back straight and supported by a chair, legs and arms uncrossed, and feet flat on the floor. They rested quietly for about 5 minutes in this position before blood pressure was measured using the OMRON HEM-907XL. This equipment allowed for a replicated automatic measure of three sequential systolic and diastolic blood pressure readings that were then averaged. A proper cuff size to fit the upper arm was used and participants were asked to remain quiet and still throughout the measurement process. Blood pressure classifications were based on the new American College of Cardiology/American Heart Association blood pressure guidelines which is as follows: Normal: < 120/80 mm Hg; Elevated: Systolic between 120–129 and diastolic less than 80; Stage 1: Systolic between 130–139 or diastolic between 80–89; Stage 2: Systolic at least 140 or diastolic at least 90 mm Hg (Whelton et al., 2018).

#### Nutrient intake and diet quality

Dietary intake was assessed using two 24-hour dietary recalls collected on nonconsecutive days. These dietary recalls were interviewer-administered using the United States Department of Agriculture’s (USDA) 5-step multiple pass method to collect detailed information on food and beverages consumed in the 24 hours preceding the study visit (Blanton, Moshfegh, Baer, & Kretsch, 2006). This approach consists of five successive passes or steps of questioning designed to help individuals recall what they consumed in detail. Food models were also used to estimate portion sizes and improve accuracy of dietary intake. The dietary recalls were analyzed using the Nutrition Data System for Research (NDSR) software developed by the University of Minnesota Nutrition Coordinating Center (NCC). The NCC Food and Nutrient database serves as the source of food composition information in NDSR and includes over 18,000 foods with 8,000 brand name products (Sievert, Schakel, & Buzzard, 1989). The two dietary recalls were used to estimate usual nutrient intakes along with the prevalence of inadequate intakes among homeless youth. Diet quality was determined using the Healthy Eating Index (HEI-2010). HEI is a validated measure of diet quality that evaluates compliance with the Dietary Guidelines for Americans (Guenther, Reedy, & Krebs-Smith, 2008). It is comprised of 12 components (9 of which should be consumed in adequate amounts and 3 that should be consumed in limited amounts), that sum to provide the total HEI score. HEI scores range from 0 to100 points. High scores indicate intakes close to recommended amounts and low scores reflect a lower compliance with recommendations (Guenther et al., 2014). Based on criteria developed from previous studies, an HEI score above 80 indicates a ‘good’ diet, a score of 51–80 implies a ‘fair’ diet that needs improvement, and a score less than 51 reflects a ‘poor’ diet (Basiotis, Carlson, Gerrior, Juan, & Lino, 2002).

### Comparison to college students

As a reference, the homeless youth data were compared to a convenient sample of 145 college students who were recruited for another study within the same metropolitan area. Data available from the cohort included anthropometric, biochemical, clinical and diet quality data assessed using methods similar to those utilized in the current study. The college student sample was between ages 18–24 years. They were mostly female (69%) with 75% being non-Hispanic White, 14% being Asian/Pacific Islander and 7% and 3% identifying as Hispanic and African American respectively.

### Statistical analysis

Sociodemographic and homeless experience characteristics of the homeless youth sample were summarized by descriptive statistics. To determine adequacy of nutrient intake of homeless youth, nutrient data from the two dietary recalls were adjusted to usual intake and compared to Estimated Average Requirements (EAR) for age and gender. The adjustments were made using the National Research Council method (IOM, 2011). This method estimates the distribution of usual intake from observed intakes and an estimate of the measurement error associated with these intakes, both between and within individuals (IOM, 2011). Estimated average requirements were calculated via a SAS macro provided by GH.Beaton (IOM, 2003) (version January 2002) in SAS software (Version 9.3 of the SAS System for Windows). Gender differences of inadequacy of intake were made by Fisher’s exact test. Comparisons of diet quality as well as anthropometric biochemical and clinical measures between homeless youth and college students were made by independent sample t-tests for continuous variables and two sample tests of proportions for categorical variables, along with 95% confidence intervals for differences between groups. Summary statistics and comparative analyses were analyzed in Stata 13.0 (StataCorp. 2013. *Stata Statistical Software: Release 13*. College Station, TX: StataCorp LP). All reported p-values and confidence intervals are two sided and reported at the 5% nominal level.

## Results

### Sample description

**Figure 1.**
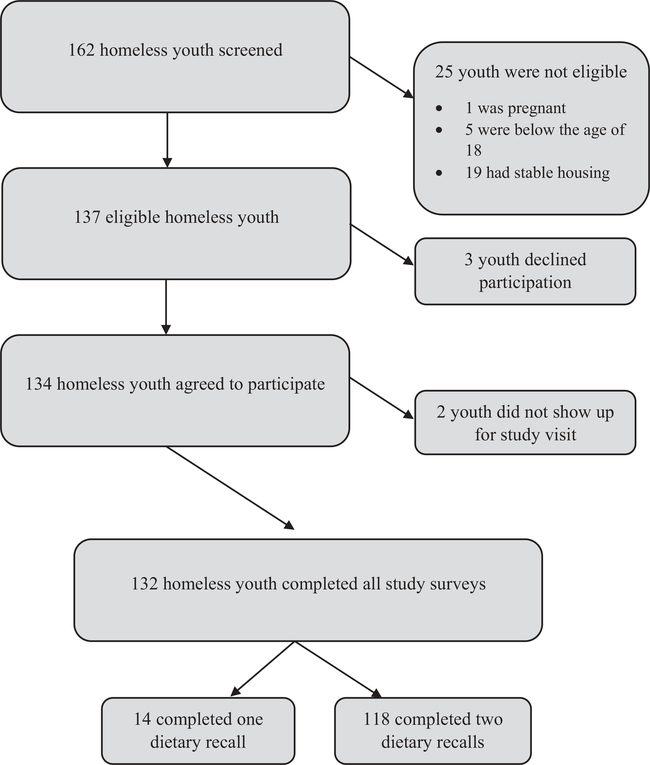
Recruitment and study completion flowchart.

Of the 162 homeless youth approached, 132 were eligible and successfully completed the study surveys. Of those eligible and consented, 118 (89%) of the participants completed two dietary recalls (Figure 1). Participants with two dietary recalls were included in the analysis because of the accuracy of multiple recalls for nutrient assessment (Thompson et al., 2015). This is especially important in a population that has difficulty with daily food access. Those who did not complete two recalls were of similar age, gender, race and education levels. Table 1 describes the sociodemographic characteristics of the homeless youth sample. Over two thirds (71%) were male with about half (51%) identifying as African-American and a third (31%) as non-Hispanic White. Majority (65%) of the youth had a high school or an equivalent General Education Diploma (GED). Only about 14% reported working more than 40 hours per week. Approximately half (47%) reported being between the age of 16–18 years the first time they were homeless. Over a third (43%) of youth have been without a home for greater than 6 months.

### Anthropometric, biochemical and clinical indicators

Mean BMI for homeless youth was 27.81 (sd = 8.31). As shown in Table 2, mean BMI of homeless youth was significantly higher compared to college students (average difference (relative to homeless youth): −3.94 (95% CI: −5.62, −2.26)). Obesity was more prevalent among homeless youth than among college student (29% vs. 8% respectively, average difference: 20.5%, 95% CI: 11.2, 29.9). Among homeless youth, 74% of females compared to 41% of males were overweight/ obese (average difference: 33.1%, 95% CI: 14.9, 51.2). Similarly, female homeless youth had a significantly higher prevalence of obesity compared to college students (59% vs 5%, average difference in proportions: −53.8%, 95% CI: −70.9%, −36.7%). Further analysis showed that 41% (n = 14) of homeless youth females were morbidly obese (classes II and III) compared to only 2% (n = 2) of college student females. Furthermore, WHtR measurements indicated that 59% of homeless youth compared to 37% of college students were at an increased risk for having cardiometabolic abnormalities. There was no significant difference among males with respects to increased cardiometabolic risk. Among females, however, 79% of homeless youth were at an increased cardiometabolic risk compared to 37% of college students (average difference in proportions: −45.4%, 95% CI: −61.8%, −28.9%).

Based on established blood pressure classification criteria (Whelton et al., 2018), 59%, 21% and 18% of homeless youth respectively had normal, elevated and high blood pressure stage 1. The proportions were 36%, 25% and 34% respectively among college students. As shown in Table 2, the mean systolic (average difference: 5.32 (95% CI: 1.51, 9.13)) and diastolic (average difference: 5.91 (95% CI: 3.11, 8.71) blood pressure levels were significantly lower among homeless youth than among college students. Similar results were observed within gender, where both male and female homeless youth had significantly lower systolic and diastolic blood pressure levels than their college student counterparts.

Mean total cholesterol concentration was significantly lower in homeless youth than in college students (average difference: 29.78 (95% CI: 19.32, 40.24). On the contrary, total triglycerides concentrations were significantly higher in homeless youth than in college students (average difference: −38.75 (95% CI: −64.44, −13.06)), with female homeless youth having significantly higher triglycerides concentrations than female college students (average difference: −39.16 (95% CI: −67.44, −10.88)).

### Nutrient intake and diet quality indicators

The average HEI score for homeless youth was 44.37 (SD: 12.64/Table 3) which is indicative of poor diet quality (Basiotis et al., 2002). Compared to college students, homeless youth had significantly lower HEI scores (average difference: 10.71 (95% CI: 7.24, 14.18). This pattern was reflected in almost all components of diet quality. Within gender, homeless females had significantly poorer intakes of total fruit (average difference: 1.36 (95% CI: 0.52, 2.20)), whole fruit (average difference: 1.51 (95% CI: 0.60, 2.42)), whole grain (average difference: 1.87 (95% CI: 0.27, 3.47)), dairy (average difference: 2.18 (95% CI: 0.71, 3.65)), seafood and plant proteins (average difference: 1.89 (95% CI: 1.01, 2.77)) than college students. Males had similar patterns, but overall smaller magnitudes of difference were noted between homeless youth and college students and homeless youth for these components. Different from females, homeless males had poorer intake of total vegetables (average difference: 0.98 (95% CI: 0.49, 1.47) and greens and beans (average difference: 1.11 (95% CI: 0.53, 1.69) compared to college males. Table 4 shows the prevalence of inadequacy of nutrients intake among homeless youth. Findings show that nearly all youth had adequate intakes of vitamins B_1_, B_2_, B_6_ and B_12_. This is not a surprising finding as we also found (Table 3) that homeless youth consumed inexpensive refined cereal-grain products, which are mandatorily fortified with these nutrients in the US (Cook & Welsh, 1987). Over 70% of the youth, however, had inadequate intakes of vitamins A, C, D_3_ and E, as well as calcium and magnesium. Specifically, over 80% of males had inadequate intakes of vitamin A and D_3_, along with calcium and magnesium. For females, over 80% had inadequate intakes of vitamin A, C, D_3,_ E, and calcium, while nearly 60% were inadequate in protein intake.

## Discussion

This study among unaccompanied homeless youth provides evidence indicating insufficient dietary and nutrients intake as well as increased weight related parameters. The poor dietary intake among homeless youth were reflected by 1) an extremely poor diet quality, devoid of health promoting foods such as fruit, vegetables and whole grains and 2) inadequate intakes in essential micronutrients such as vitamins A,C, D_3_, E calcium and magnesium. We also found obesity to be more prevalent among homeless female youth compared to non-homeless youth of similar age. The obesity prevalence of 29% in this cohort of homeless youth is also significantly higher than the national average of 17% reported among youth (Ogden, Carroll, Kit, & Flegal, 2014). The severest obesity rate was among homeless females, who were also found to be at a higher risk for cardiometabolic abnormalities. To our surprise, however, a higher proportion of homeless youth were normotensive compared to their college student counterparts.

Young adults, in general, have poor dietary quality (Delisle and WHO 2005; Demory-Luce et al., 2004), however, our findings among this cohort of homeless youth shows a much poorer dietary pattern compared to the general population of US youth (Gu & Tucker, 2017; USDA, 2018). In a separate unpublished analysis, we found the onset of homelessness to be to be the only predictor of poor dietary intake among this cohort of homeless youth. Youth who experienced homelessness at a younger age were more likely to have a poor dietary pattern. As such, the extremely poor diet quality reported by homeless youth maybe related to their state of homelessness. It is possible that the absence of a stable home, during the formative ages, when eating habits are still being developed, could be influencing their dietary patterns (Richards & Smith, 2007). The extent of poor dietary intake among homeless youth is further demonstrated by our findings of inadequate intake of several essential nutrients. While this is not a surprising finding, based on results shown among homeless youth in Toronto (Tarasuk et al., 2005), it confirms that homeless youth face challenges with the acquisition and consumption of healthy foods (Tarasuk et al., 2009; Whitbeck, Chen, & Johnson, 2006). Numerous environmental, social, and societal factors contribute to the difficulty of food acquisition experienced by these youths (Whitbeck et al., 2006). Lower-income neighborhoods, where homeless populations tend to inhabit (Alexander-Eitzman, Pollio, & North, 2013), typically have less fresh produce, less overall variety, and fresh foods often cost more, making access to healthy foods limited (Hendrickson, Smith, & Eikenberry, 2006). Moreover, food establishments that offer inexpensive but calorie-dense and highly processed foods (such as fast-food restaurants and convenience stores) are often easily accessible in these environments (Antoniades & Tarasuk, 1998; Dachner & Tarasuk, 2002; Tarasuk et al., 2005). For a population that lacks financial and food preparation resources, these poorer quality foods may be their only option (Antoniades & Tarasuk, 1998; Dachner & Tarasuk, 2002). Even when youth are able to live in homeless shelters, many shelters lack a place to store perishable food items and may only permit packaged food items while on the premises (Richards & Smith, 2007). On the other hand, some youths may simply not desire to eat healthy foods even if they had access to them. Their eating habits and preferences for particular foods are often formed in early childhood, and are therefore less likely to change during young adulthood (Birch, Savage, & Ventura, 2007).

Previous studies have investigated the prevalence of overweight and obesity among homeless youth with inconsistent findings (Cutuli et al., 2015; Tarasuk et al., 2005). Our findings show a high prevalence of overweight and obesity in this population, especially among females. The overweight and obesity prevalence rate for our sample was 50%, which is higher than those reported in other studies among homeless youth (Cutuli et al., 2015; Tarasuk et al., 2005). Unique to our study was the determination of waist-to-height ratios, which corroborated the elevated BMI results. These findings show that homeless youth are at an increased risk for developing many chronic health issues including type 2 diabetes and cardiovascular disease based on their poor weight status (Frier & Greene, 2005). The high weight and adiposity status among this group could again be explained by poor diet quality, including the consumption of foods that lead to increased adiposity (Dachner & Tarasuk, 2002; Tarasuk et al., 2005). Similarly, the high levels of stress produced by the homeless environment could also be a contributing factor to the high adiposity (Wardle, Chida, Gibson, Whitaker, & Steptoe, 2011). This explanation is especially plausible as we found obesity rates to be high among homeless females. Studies have shown a higher risk for stress among homeless females compared to males (Gwadz, Nish, Leonard, & Strauss, 2007).

Although increased weight and adiposity are both associated with increased cardiometabolic risk parameters including lipid profile and blood pressure, not all overweight or obese individuals develop high blood pressure (Zanchetti, 2015). There is evidence that their effects on cardiometabolic risk parameters are not well understood (Zanchetti, 2015). Our findings that homeless youth (who reported increased weight and adiposity) had better blood pressure values compared to college students, was unexpected and quite surprising. An explanation for this finding is not clear; our results may be due to differences in the equipment and methods used in measuring blood pressure and lipid profile.

One major strength of this current study is that, it is the first of its kind to be conducted among unaccompanied homeless youth in the US. In addition, the presence of a seemingly healthy and housed comparison group from the same city further highlights the critical needs of this group. A few limitations of the study need to be mentioned as well. First, the study involved a convenient sample of homeless youth recruited from a drop-in center in a Midwestern city in the U.S. Our findings may therefore not be generalizable to other homeless youth populations. Additionally, youth included in this study were those who chose to visit the drop-in center during the duration of our study. This introduces selection bias, since there is undoubtedly a subset of homeless youth who do not utilize drop-in centers and were therefore not represented. The study’s generalizability is also limited by the small and convenient nature of the sample of comparison college students. Furthermore, the study utilized self-reported dietary data, which introduces challenges of both under- and over-reporting, along with recall and social approval bias (Slimani, Freisling, Illner, & Huybrecht, 2015). Finally, it lacks information on substance use among homeless youth, which can affect their dietary intake.

## Implication and contribution

Unaccompanied homeless youth have poor dietary patterns with related adiposity. This aspect of their health, however, remains understudied with no consistent efforts to address their nutritional wellbeing. Our findings show nutritional vulnerabilities among unaccompanied homeless youth. Future studies are needed to inform evidence-based nutrition policies and interventions that will aid in improving their nutritional and overall health.

## Figures and Tables

**Table 1.** Sociodemographic and homeless experience characteristics of homeless youth, n (%).

Characteristic	Males (n = 84)	Females (n = 34)	Total sample (n = 118)
Age, y *Mean (sd)*	21.4 (1.7)	21.4 (1.9)	21.4 (1.8)
18–20	24 (29)	11 (32)	35 (30)
21–24	60 (71)	23 (68)	83 (70)
Race/Ethnicity			
White non-Hispanic	24 (29)	9 (26)	33 (28)
African-American/Black non-Hispanic	36 (43)	18(53)	54 (46)
Other (Asian, Latin American & Hispanic)	24 (29)	7(21)	31 (26)
Education			
Grade≤ 9	7 (8)	2 (6)	9 (8)
Grade 10–11	25 (30)	7 (21)	32 (27)
Grade ≥12	52 (62)	25 (74)	77 (65)
Employment status			
Work 40+ hr/wk	13 (15)	4 (12)	17 (14)
Fewer than 40 hr/wk	16 (19)	6 (18)	22 (19)
Student	5 (6)	4 (12)	9 (8)
Unemployed	50 (60)	20 (59)	70 (63)
Age at first homelessness			
≤ 15 yrs	12 (14)	2 (6)	14 (12)
16–18 yrs	38 (45)	18 (53)	56 (47)
≥19 yrs	34 (40)	14 (41)	48 (41)
Length of homelessness			
< 1 month	26 (31)	10 (29)	36 (31)
1–6 months	20 (24)	11 (32)	31 (26)
> 6 months	38 (45)	13 (38)	51 (43)
Perceived health status Very good/excellent	34 (40)	10 (29)	44 (37)
Good	27 (32)	12 (35)	39 (33)
Fair/poor	23 (27)	12 (35)	35 (30)

**Table 2.** Comparison of anthropometric, biochemical and clinical indicators associated with nutrition status in college students and homeless youth.

Measure	College Students Mean (SD)			Elomeless Youth Mean (SD)			Mean Difference (95% Cl)^a^	
	Males (n = 45)	Females (n = 100)	All (n = 145)	Males (n = 84)	Females (n = 34)	All (n = 118)	Male	Female
BMI^b^	25.43 (3.99)	23.17 (4.51)	23.87 (4.47)	25.91 (6.76)	32.51 (9.91)	27.81 (8.31)	−0.48 (−2.36, 1.39)	−9.34 (−12.90, −5.78)
Overweight	12 (26.7%)	16 (16.0%)	28 (19.3%)	20 (23.8%)	5 (14.7%)	25 (21%)	2.9% (−12.9%, 18.7%)	1.3% (−12.6%, 15.2%)
Obese	7 (15.6%)	5 (5.0%)	12 (8.3%)	14 (16.7%)	20 (58.8%)	34 (29%)	−1.1% (−14.4%, 12.2%)	−53.8% (−70.9%, −36.7%)
Waist circumference (cm)	89.85 (10.10)	81.59 (9.93)	84.15 (10.66)	90.52 (15.85)	99.53 (22.47)	93.11 (18.37)	−0.67 (−5.21, 3.87)	−17.94 (−26.00, −9.88)
Waist to height ratio	0.51 (0.06)	0.49 (0.06)	0.50 (0.06)	0.51 (0.09)	0.60 (0.14)	0.54 (0.11)	0 (−0.03, 0.03)	−0.11 (−0.16, −0.06)
Elevated waist to height ratio^c^	20 (44.44%)	34 (34.00%)	54 (37.24%)	43 (51.19%)	27 (79.41%)	70 (59.32%)	−6.8% (−24.8%, 11.2%)	−45.4% (−61.8, −28.9%)
Systolic blood pressure^d^	130.36 (9.93)	119.40 (9.78)	122.82 (11.04)	119.78 (13.89)	112.95 (10.14)	117.5 (13.08)	10.58 (5.30, 15.86)	6.45 (1.38, 11.52)
Diastolic blood pressure^d^	79.25 (8.65)	74.15 (7.86)	75.74 (8.42)	70.10 (9.68)	69.30 (9.28)	69.83 (9.48)	9.15 (5.17, 13.13)	4.85 (0.28, 9.42)
Triglycerides mg/dL^e^	70.45 (48.69)	65.51 (26.99)	67.05 (35.07)	106.41 (112.86)	104.67 (60.01)	105.80 (97.08)	−35.96 (−75.07, 3.15)	−39.16 (−66.91, −11.41)
HDL cholesterol mg/dL ^e^	43.56 (9.67)	53.07 (12.58)	50.12 (12.52)	52.38 (18.08)	50.57 (17.83)	51.75 (17.87)	−8.82 (−15.30, −2.34)	2.50 (−5.93, 10.93)
Total cholesterol mg/dL ^e^	174.96 (31.49)	181.83 (31.76)	179.70 (31.73)	148.13 (37.28)	153.24 (32.17)	149.92 (35.38)	26.83 (11.70. 41.96)	28.59 (12.83. 44.35)

a Mean difference and 95% Cl comparing college students and homeless youth by gender

b Body Mass Index

c Elevated defined as greater than global boundary of 0.5 for both males and females

d Blood pressure data was available for only 60 homeless youth, 40 males, 20 females

e Lipid panel data was available for only 60 homeless youth, 39 males, 21 females

**Table 3.** Comparison of diet quality in college students and homeless youth.

HEI- 2010 Dietary Component	Maximum score	College Students Mean (SD)			Elomeless Youth Mean (SD)			Mean Difference (95% Cl) ^a^	
		Males (n = 45)	Females (n = 100)	All (n = 145)	Males (n = 83)	Females (n = 34)	All (n = 117)	Males	Females
Total vegetables^b^	5	2.72 (1.80)	2.86 (1.75)	2.82 (1.76)	1.74 (1.54)	2.51 (1.63)	1.96 (1.60)	0.98 (0.35, 1.61)	0.35 (−0.31, 1.01)
Greens and beans^b^	5	1.98 (2.19)	1.83 (2.29)	1.87 (2.25)	0.87 (1.82)	1.08 (2.04)	0.93 (1.88)	1.11 (0.35, 1.87)	0.75 (−0.09, 1.59)
Total fruit^b^	5	1.62 (1.99)	2.62 (2.02)	2.31 (2.06)	1.26 (1.76)	1.38 (1.99)	1.29 (1.82)	0.36 (−0.34, 1.06)	1.36 (0.64, 2.08)
Whole fruit^b^	5	1.95 (2.10)	2.89 (2.24)	2.60 (2.23)	0.90 (1.64)	0.85 (1.59)	0.89 (1.62)	1.05 (0.33, 1.77)	1.51 (0.72, 2.30)
Whole grain^b^	10	3.41 (3.91)	4.27 (3.67)	4.00 (3.76)	2.01 (2.90)	2.40 (3.42)	2.12 (3.05)	1.40 (0.08, 2.72)	1.87 (0.49, 3.25)
Dairy^b^	10	5.87 (3.66)	6.01 (3.32)	5.97 (3.42)	4.54 (3.11)	3.83 (3.19)	4.33 (3.14)	1.33 (0.05, 2.61)	2.18 (0.90, 3.46)
Total protein foods^b^	5	4.33 (1.36)	4.04 (1.59)	4.13 (1.52)	3.95 (1.74)	3.65 (1.51)	3.86 (1.68)	0.38 (−0.17, 0.93)	0.39 (−0.22, 1.00)
Seafood & plant proteins^b^	5	1.72 (2.25)	2.66 (2.35)	2.37 (2.35)	1.06 (1.80)	0.77 (1.58)	0.97 (1.67)	0.66 (−0.12, 1.44)	1.89 (1.18, 2.60)
Fatty acids^b^	10	5.17 (3.75)	4.58 (3.81)	4.76 (3.79)	5.40 (3.41)	6.59 (3.65)	5.74 (3.51)	-0.23 (−1.57, 1.11)	-2.01 (−3.48, −0.54)
Sodium^c^	10	4.39 (3.00)	3.88 (3.47)	4.04 (3.33)	3.42 (3.68)	4.05 (3.65)	3.60 (3.67)	0.97 (−0.22, 2.16)	-0.17 (−1.60, 1.26)
Refined grains^c^	10	4.19 (3.76)	5.53 (4.03)	5.12 (3.99)	4.75 (3.76)	5.28 (3.83)	4.90 (3.77)	-0.56 (−1.94, 0.82)	0.15 (−1.39, 1.69)
Empty calories^c,d^	20	15.39	14.96	15.06	14.20	13.83	14.09	1.19	1.13
		(4.14)	(5.10)	(4.82)	(5.13)	(6.00)	(5.37)	(−0.47, 2.85)	(−1.10, 3.36)
**Total HEI Score**	**100**	**52.75**	**56.13**	**55.08**	**43.84**	**45.65**	**44.37**	**8.91**	**10.48**
		**(15.09)**	**(16.14)**	**(15.84)**	**(12.46)**	**(13.17)**	**(12.64)**	**(3.67, 14.15)**	**(4.94, 16.02)**

a Mean difference and 95% Cl comparing college students and homeless youth by gender

b Components to be consumed in adequate amounts for which higher score indicates higher consumption

c Components to be consumed in moderate amounts for which higher score indicates lower consumption

d Empty calories from solid fats, alcohol (intake above 13g/1 OOOkcal) and added sugars.

**Table 4.** Estimated usual intake of nutrients and prevalence of intake inadequacy among homeless youth.

Nutrient	Males (n = 84)		Females (n = 34)	
	Usual Intake *mean (sd)*	% Inadequate *n(%)*	Usual Intake *mean (sd)*	% Inadequate *n(%)*
Protein, (g/kg/d)	1.07 (0.66)	28 (33.3%)	0.83 (0.62)	20 (58.8%) ^a^
^b^Vitamin A, μg/d	236.73 (174.72)	70 (83.3%)	365.19 (274.67)	30 (88.2%)
Thiamin, mg/d	0.91 (0.34)	0 (0)	0.92 (0.36)	0 (0)
Riboflavin, mg/d	1.05 (0.70)	0 (0)	0.95 (0.37)	0 (0)
Niacin, mg/d	21.53 (13.89)	1 (1.2%)	18.93 (5.99)	4 (11.8%) ^a^
Vitamin B_6_, mg/d	1.11 (1.97)	0 (0)	0.98 (0.45)	0 (0)
^c^Folate, μg/d	292.95 (233.68)	8 (9.5%)	318.89 (234.65)	15 (44.1%) ^a^
Vitamin B_12_, μg/d	2.49 (2.45)	1 (1.2%)	2.15 (1.62)	1 (2.9%)
Vitamin C, mg/d	33.16 (34.38)	60 (71.4%)	43.77 (44.19)	30 (88.2%)
Vitamin D_3_, μg/d	2.00 (1.66)	72 (85.7%)	1.99 (2.00)	30 (88.2%)
^d^Vitamin E, mg/d	3.63 (2.91)	64 (76.2%)	4.10 (2.04)	28 (82.4%)
Calcium, mg/d	388.77 (168.85)	72 (85.7%)	369.80 (145.01)	27 (79.4%)
Iron, mg/d	7.19 (4.00)	1 (1.2%)	8.49 (7.14)	9 (26.5%) ^a^
Magnesium, mg/d	105.00 (24.83)	72 (85.7%)	120.79 (55.20)	25 (73.5%)
Phosphorus, mg/d	521.14 (131.62)	10 (11.9%)	524.35 (156.67)	14 (41.2%) ^a^
Selenium, μg/d	56.00 (17.94)	6 (7.1%)	53.49 (16.23)	9 (26.5%) ^a^
Copper, μg/d	464.58 (138.36)	28 (33.3%)	554.51 (304.23)	15 (44.1%)
Zinc, mg/d	5.22 (2.70)	35 (41.7%)	5.00 (2.53)	12 (35.3%)

a Fisher’s exact test comparing proportion of males and female with inadequacy p-value < 0.05.

b As retinol activity equivalents

c As dietary folate equivalents

d As α-tocopherol
